# Engineered cells as glioblastoma therapeutics

**DOI:** 10.1038/s41417-021-00320-w

**Published:** 2021-03-22

**Authors:** Aparna Ramanathan, Ian A. J. Lorimer

**Affiliations:** 1 Cancer Therapeutics Program, Ottawa Hospital Research Institute, Ottawa, Canada; 2 Department of Medicine, University of Ottawa, Ottawa, Ontario Canada; 3 Department of Biochemistry, Microbiology and Immunology, University of Ottawa, Ottawa, Ontario Canada

**Keywords:** CNS cancer, Drug development

## Abstract

In spite of significant recent advances in our understanding of the genetics and cell biology of glioblastoma, to date, this has not led to improved treatments for this cancer. In addition to small molecule, antibody, and engineered virus approaches, engineered cells are also being explored as glioblastoma therapeutics. This includes CAR-T cells, CAR-NK cells, as well as engineered neural stem cells and mesenchymal stem cells. Here we review the state of this field, starting with clinical trial studies. These have established the feasibility and safety of engineered cell therapies for glioblastoma and show some evidence for activity. Next, we review the preclinical literature and compare the strengths and weaknesses of various starting cell types for engineered cell therapies. Finally, we discuss future directions for this nascent but promising modality for glioblastoma therapy.

## Introduction

Glioblastoma is the most common malignant primary brain tumor, with an incidence of 1–4 per 100,000 [1]. The past decade has seen significant advances in our knowledge of glioblastoma pathophysiology. This includes detailed studies of genetics [2], epigenetics, and expression profiling [3] at the bulk- and single-cell level [4] in large patient cohorts and in longitudinal studies [5], as well as barcoding studies in mouse models to understand glioblastoma cell hierarchies and plasticity [6, 7]. In addition, recent studies have demonstrated long-distance physical connections between glioblastoma cells and connections between glioblastoma cells and neighboring normal cells [8–10]. These findings reinforce our understanding of glioblastoma as a highly heterogeneous cancer that exists as an almost organ-like structure distributed through much of the brain. In spite of these advances, glioblastoma remains an incurable disease with median survival after diagnosis of about 15 months [11]. Frustratingly, therapies that have improved outcomes in other cancers have been ineffective in glioblastoma. Examples of this are the small molecule EGFR tyrosine kinase inhibitors, which enhance survival in lung cancer but are ineffective in glioblastoma [12, 13], even though *EGFR* is amplified and mutated to a constitutively active form in many glioblastomas. Similarly, immune checkpoint inhibitors have also had success in other cancers, particularly melanoma, but again have been ineffective in glioblastoma [14], outside of a recent small trial of a PD-1 inhibitor administered before second surgeries [15]. Thus while we have a much-improved understanding of glioblastoma, to date this has not led to substantial improvements in treatment. The unique and complex features of this disease suggest that new therapeutic modalities may be needed to develop truly effective treatments. Although small molecules and antibodies are currently by far the most common therapeutic modalities in the cancer field, engineered viruses and cells are also being developed as therapeutics. These potentially have very sophisticated capabilities that we have only begun to explore. Virus-based therapies for glioblastoma have been reviewed elsewhere [16]. The focus of this review is on the use of engineered cells to treat glioblastoma, with an emphasis on data from the small number of clinical trials that have been performed to date and the lessons learned from these.

## Clinical data on engineered cells for glioblastoma therapy

At this time there are six publications describing clinical studies that evaluate engineered cells as glioblastoma therapeutics. Five of the six publications involve engineered T cells, while one involves the use of engineered neural stem cells. Details of these are described in the following sections.

### Engineered T cells

Chimeric antigen receptor (CAR)-T cells are by far the most established engineered cell therapy for cancer [17]. Multiple countries have now approved their use in treating some types of lymphoma and leukemia [18], based on their ability to induce remissions that are, in some patients, very durable. The basic process for CAR-T-cell production is to isolate T cells from a patient, expand them ex vivo, genetically modify them to express an engineered T-cell receptor, and then inject them back into the same patient. Patients are generally lymphodepleted prior to administration of CAR-T cells. Key to the technology is the design of the engineered T-cell receptor [17]. In all formats, the extracellular domain of the receptor, which normally recognizes a peptide presented by MHC, is replaced by an alternate recognition domain. Most commonly, single-chain Fvs are used for this purpose, although other domains such as engineered receptor ligands are also being explored. Intracellularly, the CAR contains the CD3ζ signaling domain, which contains ITAM motifs capable of recruiting downstream signaling molecules. This format replaces the normal, multichain T-cell receptor complex with a single protein. The nature of the hinge region connecting the new targeting domain to the receptor and the nature of the transmembrane domain are also critical design factors. This outlines the basics of a so-called “first generation” design: second and third-generation designs include, respectively, one or two intracellular costimulatory domains, most commonly derived from 4-1BB (CD137) and CD28 proteins. These enhance both initial potency and the persistence of T cells after administration. Ex vivo enrichment of less differentiated T cells with greater regenerative potential is a second strategy to being explored promote T-cell persistence. Activation of CAR-T cells after antigen engagement results in cytokine release, T-cell proliferation, and directed exocytosis (degranulation) of perforin and granzymes, leading to destruction of the antigen-bearing cell. While CAR-T cells have been remarkably successful in some lymphomas and leukemias, the application of this technology to solid tumors has so far only met with limited success. The following summarizes current clinical experience with CAR-T cells in glioblastoma.

The first report of a clinical trial of CAR-T cells in glioblastoma was published in 2015 [19]. This trial evaluated a first-generation CAR targeted the interleukin-13 receptor alpha 2 (IL13Rα2) on glioblastoma cells. Rather than using a single-chain Fv targeting the receptor, targeting was accomplished using the receptor ligand, IL13, that was mutated to have improved selectivity for IL13Rα2 [20]. Engineered CD8+ T cells were administered into the resection cavity. Details of this trial and other trials discussed below are summarized in Table 1. Key takeaways from this trial were that the treatment was well-tolerated and showed hints of activity, with decreased IL13Rα2 expression and increased necrosis in one patient that had high initial target expression. Lack of T-cell persistence and antigen heterogeneity were viewed as probable significant limitations.

**Table 1:** Clinical data on engineered cells for glioblastoma therapy.

Reference(s)	Brown et al. (2015) *Clin Cancer Res*, 21(18), 4062–4072.	Brown et al. (2016) *N Engl J Med*, 375(26), 2561–2569.	O’Rourke et al. (2017) *Sci Transl Med*, 9(399).	Ahmed et al. (2017) *JAMA Oncol*, 3(8), 1094–1101.	Goff et al. (2019) *J Immunother*, 42(4), 126–135.	Portnow et al. (2017) *Clin Cancer Res*, 23(12), 2951–2960.
Title	Bioactivity and safety of IL13Rα2-redirected chimeric antigen receptor CD8+ T cells in patients with recurrent glioblastoma	Regression of glioblastoma after chimeric antigen receptor T-cell therapy	A single dose of peripherally infused EGFRvIII-directed CAR-T cells mediates antigen loss and induces adaptive resistance in patients with recurrent glioblastoma	HER2-specific chimeric antigen receptor-modified virus-specific T cells for progressive glioblastoma: a phase 1 dose-escalation trial	Pilot trial of adoptive transfer of chimeric antigen receptor-transduced T cells targeting EGFRvIII in patients with glioblastoma	Neural stem cell-based anticancer gene therapy: a first-in-human study in recurrent high-grade glioma patients
Type of cell used	Autologous CD8+ T cells	Autologous central memory T cells	Autologous T cells	Autologous virus-specific T cells	Autologous peripheral blood lymphocytes	HB1.F3 neural stem (NSC) cell line
Modifications to cells	Electroporated PBMC with IL13Rα2-selective ligand-based CAR (IL13 E13Y-mutated) with CD3ζ signaling domain	Lentivirally transduced T cells with IL13Rα2-selective ligand-based CAR (IL13 E13Y-mutated) with CD3ζ signaling domain and 4-1BB costimulatory domain	Lentivirally transduced T cells with CAR with humanized EGFRvIII-specific scFv, CD3ζ signaling domain and 4-1BB costimulatory domain	Adenoviral transduction of PBMC with CMV pp65 antigen. Retroviral transduction of virus-specific T cells with CAR with murine HER2-specific scFv, CD3ζ signaling domain and 4-1BB costimulatory domain	Retrovirally transduced with CAR with human EGFRvIII-specific scFv, CD3ζ signaling domain, CD28 and 4-1BB costimulatory domains	Retrovirally transduced NSC with *v-myc* and cytosine deaminase (CD) gene
Route of administration	Intracavitary	Intracavitary Intraventricular	Intravenous	Intravenous	Intravenous	Intracranial administration into wall of the resection cavity or tumor tissue
Number of patients	3 patients	1 patient	10 EGFRvIII+ patients	17 HER2+ patients	18 EGFRvIII+ patients	15 high-grade glioma patients
Number of cells delivered	Dose escalation: 12 doses of 10^7^ –10^8^ cells	Intracavitary: 2 × 10^6^ cells initial infusion, 5 infusions of 1 × 10^7^ cells Intraventricular: 2 × 10^6^ cells initial infusion, 9 infusions of 1 × 10^7^ cells	Median dose: 5 × 10^8^ cells Range: 1 × 10^8^–5 × 10^8^ cells	Dose escalation: 1 or more infusions at 5 dose levels: 1 × 10^6^/m^2^, 3 × 10^6^/m^2^, 1 × 10^7^ /m^2^, 3 × 10^7^/m^2^, and 1 × 10^8^/m^2^	Dose escalation: 10^7^ to ≥10^10^ cells	Dose escalation: 1 × 10^7^ or 5 × 10^7^
Persistence of cells	Low levels of persistence after 14 weeks	No data for intracavity infusions. After intraventricular therapy, cells were detected in CSF for ≥7 days. Small increase in number of CAR-T cells in CSF 2 days after infusion, followed by a gradual decrease. When tumor burden was lower (in later cycles), the number of CAR-T cells in	Peripheral blood: CAR-T-cell peak expansion occurred between days 3 and 10. After day 14, levels of circulating cells rapidly declined with none detected after 30 days. Tumor: CAR-T cells detected in 5 of 7 patients. In two, levels were higher than in blood.	Cells did not expand but were present in peripheral blood up to 12 months after infusion in some samples. Expansion at tumor sites suggested by pseudoprogression seen on MRI in 5 patients	Persistence in peripheral blood detected in 13 of 14 patients at 1 month, in 5 patients at 3 months.	Small number of CD-NSCs remained in the brain 3 months after treatment (from autopsy data), present as single, non-dividing cells in tumors.
Evidence for activity	Reduced IL13Rα2 expression in tumor tissue, increased necrosis	Intracavitary infusions: Treated tumor was stable for >45 days. Nonresected tumors and new tumors progressed. New metastatic lesions in spine detected. Intraventricular infusions: Complete regression of metastatic tumors in spine. Clinical response sustained for 7.5 months	EGFRvIII expression decreased in 5 of 7 patients, undetectable in 2 after treatment	One patient with partial response that was sustained for 9 months, 7 with stable disease by MRI assessment	No objective responses based on MRI imaging	Conversion of 5-FC: 5-FC-dose-dependent increase in 5-FU observed in brain extracellular fluid. Plasma 5-FU concentrations lower than brain interstitial 5-FU concentrations. NSC migration to tumors: v-myc-positive areas detected within distant tumor foci
Immune responses	Inflammation at injection site detected by MRI	Immediate increase in endogenous immune cells after intraventricular infusion: endogenous CD3+ T cells, CD19+ B cells, CD14+CD11b+HLA-DR mature myeloid populations, CD11b+CD15+ granulocytes. Inflammatory: Elevated levels of cytokines were also present in the CSF, including interferon-γ, TNF-α, interleukins 2, 10, 5, 6, and 8; chemokines CXCL9, CXCL10; and CCR2; IL-1 receptor α. No significant increase in cytokine and CAR-T-cell levels detected in peripheral blood. Cytokine levels returned to baseline levels between weekly treatments.	Infiltration of endogenous T cells as well as CAR-T cells. Increased expression of immunosuppressive molecules IDO1, FoxP3, and IL10. Increase in proportion of FoxP3 + CD3 cells.	Possible local T-cell expansion and local inflammatory responses detected by MRI (pseudoprogression)		No anti-NSC antibodies were detected at any time.
Treatment-related adverse events	Headache, one transient grade 3 neurologic event	Grade 1 or 2 events: headaches, generalized fatigue, myalgia, olfactory auras.	No dose-limiting toxicities. Neurological events.	No dose-limiting toxicity was observed. 2 patients had grade 2 seizures and/or headaches	Acute dyspnea and oxygen desaturation in 2 patients at highest dose, 1 patient expired. Grade 2 neurologic symptoms or suspected seizure activity in 10 patients.	No toxicities associated with intracranial administration of CD-NSCs. 1 dose-limiting toxicity possibly due to 5-FC occurred in a patient on dose level 3 (5 × 10^7^ cells). Other toxicities possibly related to 5-FC: grade 3 fatigue, lymphopenia, thrombocytopenia

This study on the bioactivity and safety of this CAR-T-cell therapy was followed up with a case report from the same group describing a patient that showed marked regression with treatment [21]. This study used a similar strategy as above, except that the CAR was made in a second-generation format that included the 4-1BB costimulatory domain and was expressed in enriched central memory T cells. While intracavity administration was used initially, this was switched to intraventricular administration after six cycles. This was based on some evidence for tumor control in the vicinity of the injection site but not at distant sites in this patient with multifocal leptomeningeal disease. With the switch to intraventricular delivery, the patient underwent a complete response, with both intracranial and spinal tumors no longer detectable by MRI. This was sustained for 7.5 months after which disease recurred at new locations. CAR-T cells were found to persist for at least 7 days. They also increased the populations of endogenous immune cells in the CSF (B cells, T cells, and myeloid cells) and increased the CSF expression of inflammatory cytokines, which may have contributed to the response. Key takeaways from this study are the first solid evidence of clinical activity of a CAR-T-cell therapy in glioblastoma (albeit in a single patient), the apparent superiority of the intraventricular route of administration and the fact that a complete response was seen in spite of non-uniform target expression.

In 2017, results of a phase I trial of CAR-T cells targeting the mutant EGF receptor EGFRvIII in glioblastoma were reported [22]. This trial included ten EGFRvIII+ glioblastoma patients. In contrast to the above studies, CAR-T cells were administered intravenously. Key findings were that, for seven patients in which a post-treatment surgery was performed, CAR-T cells were detectable in the tumors of five of these. Levels of EGFRvIII expression were also reduced in five of seven patients, suggesting activity. As with the case report above, there was evidence for recruitment of endogenous T cells. However, additional analyses suggested that substantial numbers of these were immunosuppressive regulatory T cells. There was also an increase in immunosuppressive molecules such as IDO1 and IL10 in tumors post-treatment. This suggests an immunosuppressive response to CAR-T cells within tumors that could limit their activity.

Another study, also published in 2017, reported on a clinical trial of CAR-T cells targeting HER2 in glioblastoma [23]. Aside from the different target, a key distinguishing feature of this study was the use of virus-specific T cells. This is a strategy to promote T-cell persistence through stimulation of endogenous T-cell receptors by viral antigens [24]. A second-generation CAR with a CD28 costimulatory domain was used (a third-generation version with both CD28 and 4-1BB costimulatory domains had previously raised some safety concerns). A total of seventeen patients were treated. Infused cells did not expand in peripheral blood but could still detected 12 months after infusion in some samples. Pre- and post-treatment samples were not analyzed for changes in HER2 levels or other pharmacodynamic markers, but MRI changes in five patients were suggestive of endogenous T-cell expansion at the tumor site. MRI also showed a partial response in one patient and stable disease in seven other patients, although these changes cannot be definitively attributed to the treatment.

In 2019, Goff et al. published a pilot trial that also looked at CAR-T cells targeting EGFRvIII in glioblastoma [25]. This trial evaluated a third-generation CAR in 18 EGFRvIII+ glioblastoma patients. Patients were lymphodepleted with chemotherapy prior to CAR-T-cell infusion and supported with interleukin-2 after infusion. Doses of up to 10^10^ CAR-T cells were tested. Dose-limiting toxicities were evident at the highest dose. Persistence was dose-dependent and was higher than the previously reported EGFRvIII-targeted CAR-T-cell trial [22]. However, no objective responses were observed based on MRI assessments.

### Engineered neural stem cells

While most clinical trial activity with engineered cells as glioblastoma therapeutics has focussed on engineered T cells, one clinical trial has explored the use of engineered neural stem cells for glioblastoma therapy [26]. Like T cells, neural stem cells have an intrinsic ability to home to sites of damage and to tumors [27, 28]. Unlike T cells, neural stem cells do not have intrinsic cytotoxic activity. In this trial, a human neural stem cell line was engineered for cytotoxic activity by retroviral transduction with cDNA encoding the pro-drug activating enzyme cytosine deaminase. Cells were administered intracranially to 15 patients; this was followed by oral administration of the pro-drug 5-fluorocytosine. No toxicities related to the neural stem cells were evident, although there appeared to be some toxicities with high doses of the pro-drug. Intracerebral microdialysis experiments gave strong evidence that the administered cells were able to convert pro-drug in the brain and were able to do so over the 7-day period in which pro-drug was given. Neural stem cells were not detected in the systemic circulation but were detected in small numbers at 44 and 79 days after administration in the brains of two patients that underwent autopsies. Detailed analysis of these two patients showed the presence of neural stem cells at sites distant from the injection site, including the opposite hemisphere.

## Preclinical data on engineered cells for glioblastoma therapy

While clinical data on engineered cells as glioblastoma therapeutics is limited to engineered T cells and neural stem cells, natural killer cells and mesenchymal stem cells have also been explored in preclinical experiments. The following section describes current areas of preclinical development with these four cell types.

### Engineered T cells

The focus of this review is engineered cell therapies specifically as applied to glioblastoma. General advances in T-cell engineering for therapeutic applications have been reviewed elsewhere [17] and are not covered here. As described above, IL13Rα2, EGFRvIII, and HER2-targeted CAR-T cells have been evaluated in clinical trials in glioblastoma. CAR-T cells targeted to EphA2 [29], CD133 [30–32], and CD70 [33] have shown activity in animal models of glioblastoma. CAR-T cells that target chondroitin sulfate proteoglycan 4 have shown activity in cell culture [34]. The activity of these as single agents or in combination with CAR-T cells against other targets will need to be evaluated in glioblastoma trials.

### Engineered NK cells

Engineered natural killer (NK) cells are also being explored as glioblastoma therapeutics [35]. Like T cells, NK cells have an important role in cancer immunosurveillance [36]. Activation of their cytotoxic activity is mediated by a complex balance of activating and inhibitory signals combined with cytokine preactivation [37]. Cancer cells frequently downregulate class I MHC to evade detection by T cells; NK cells can directly kill target cells missing class I MHC, as this removes a key inhibitory signal mediated by multiple NK cell surface receptors. Cancers cells can also express activating receptor ligands such as MICA, a distant homolog of class I MHC that is upregulated by cellular stresses such as DNA damage [38, 39]. NK cells also contribute to the adaptive immune response by secreting cytokines that recruit and prime dendritic cells, which can then go on to present antigen to T cells [36]. Finally, NK cells are also effector cells for antibody-dependent cellular cytotoxicity, in which antibody bound to target cells is recognized by Fcγ receptors on NK cells. As with T cells, the normal cytotoxic functions of NK cells can be redirected and enhanced with chimeric antigen receptors. This has been shown to be the case using either the same chimeric antigen receptors used for T cells or chimeric antigen receptors modified for improved NK cell function [35]. CAR-NK cells have been constructed using either donor-derived NK cells or human NK cell lines; for the latter, the NK-92 cell line has been widely used [35, 37, 40]. CAR-NK cells have been generated that are active against many of the same targets that have been used for CAR-T cells. For glioblastoma-relevant targets, this includes EGFRvIII [41, 42], EGFRvIII/EGFR [43, 44], HER2 [45, 46], and CD133 [47]. CAR-NK cells targeting EGFRvIII, EGFR/EGFRVIII and HER2 have been shown to be active in orthotopic mouse xenograft models [43, 46, 48]. A clinical trial in glioblastoma patients of HER2-targeted CAR-NK cells generated by lentiviral transduction of the NK-92 cell line (CAR2BRAIN [35] NCT03383978) is scheduled for completion in 2022.

### Engineered neural stem cells

The clinical trial of engineered neural stem cells in glioblastoma described above made use of the HB1.F3 cell line, which was originally derived from human fetal brain tissue by v-myc immortalization [49]. This line has been characterized in detail in preclinical models with respect to tumor-homing ability [50], persistence [51, 52], and lack of tumorigenicity [53]. Additional therapeutic payloads beyond cytosine deaminase have also been explored using HB1.F3 [54]. A limitation of HB1.F3 is that it probably is not suitable for use in all patients for immunologic reasons: in the clinical trial, patients with evidence of immunogenicity towards class I or class II HLA antigens expressed by HB1.F3 were excluded [26]. Neural stem cells from other sources have also been explored for glioblastoma therapy. While neural stem cells derived from induced pluripotent stem cells are currently problematic given their propensity to form teratomas [55], neural stem cells derived by direct reprogramming of fibroblasts have been tested as glioblastoma therapeutic agents in preclinical models. Bago et al. showed that they had tumor-homing abilities and could prolong survival in mouse xenograft models when used to deliver the pro-apoptotic protein TRAIL [28]. This advances the possibility of generating neural stem cells for glioblastoma therapy from individual patients, albeit with many challenges for reliable and timely manufacturing. Technologies to derive neural stem cells by direct reprogramming of blood cells, which can be easily and abundantly obtained from patients, may facilitate this [56]. While the focus of this proposal is on glioblastoma applications, neural stem cells are also under active exploration as tools for regenerative medicine. Multiple clinical trials have studied the safety and persistence of neural stem cells from different sources [57–62] (Table 2) and this can help inform studies in glioblastoma.

**Table 2:** Clinical trials of neural stem cells in diseases other than cancer.

Reference(s)	Kalladka et al. (2016) *Lancet, 388*(10046), 787–796. Sinden et al. (2017). *Stem Cells Dev, 26*(13), 933–947.	Mazzini et al. (2015) *J Transl Med, 13*, 17.	Feldman et al. (2014). *Ann Neurol, 75*(3), 363–373. Chen et al. (2016). *Ann Neurol, 79*(3), 342–353.	Gupta et al. (2012) *Sci Transl Med, 4*(155), 155ra137.
Disease	Chronic ischemic stroke [1]	ALS [2]	ALS [3]	Pelizaeus-Merzbacher disease [4]
Type of cell used	CTX0E03—human neural stem cell line derived from human somatic stem cells	Human neural stem cells generated from human fetal brain tissue specimens	NSI-566RSC is an established neural progenitor cell line generated from fetal spinal cord tissue	Human central nervous system stem cells (HuCNS-SCs) are multipotent neural stem cells (CD133−, nestin-, and Sox2-positive)
Modifications to cell	Human somatic stem cells were genetically modified with c-mycER^TAM^, a conditional immortalizing gene, to generate the neural stem cell line CTX0E03. These cells proliferate in the presence of 4-hydroxy-tamoxifen (4-OHT), and undergo growth arrest and differentiation in the absence of 4-OHT. CTX ‘Drug Product’ (CTX-DP) is composed of CTX cells at *P* < 37 [5]	None	None	None
Route of administration	Intracerebral implantation, targeted to the putamen	Unilateral or bilateral injections into lumbar spinal cord	Unilateral or bilateral intraspinal injections into lumbar or cervical spinal cord	Cells injected into the anterior and posterior frontal centrum semiovale or corona radiata
Number of patients	11 patients divided into 4 cohorts to which increasing doses of cells were administered.	6 patients	12 patients	4 patients
Number of cells delivered	1st cohort: 2 × 10^6^ 2nd cohort: 5 × 10^6^ 3rd cohort: 1 × 10^7^ 4th cohort: 2 × 10^7^	3X microinjections of 7.5 × 10^5^ cells per injection site (unilateral = 2.25 × 10^6^ total cells, bilateral = 4.5 × 10^6^ total cells)	Final cell dose range: 5 × 10^5^ (5 unilateral injections)–1 × 10^6^ (10 bilateral injections)	7.5 × 10^7^ cells administered to 4 frontal lobe sites (3 × 10^8^ total brain dose)
Persistence of cells	Only tested in animal models. CTX cells 12 months post implantation were not proliferative. Long-term treatment of CTX-implanted animals with tamoxifen had no impact on CTX cell survival or proliferation.	Cells injected into immunodeficient nude mice were present at 6 months after implantation. Only few cells remained positive for Ki67 suggesting low levels of proliferation. Engrafted cells differentiated into neuronal cells expressing βTubulinIII antigen and astrocytes expressing glial fibrillary acidic protein.	Nests of live cells representative of the transplanted NPCs were found in the regions targeted by the transplants in post-mortem spinal cord tissue. Donor-specific DNA detected by qPCR in all patients at autopsy, suggesting persistence of cells. No tumor formation was evident [6].	

### Engineered mesenchymal stem cells

Human mesenchymal stem cells also have the ability to migrate towards glioblastoma tumors in animal models, suggesting their potential for engineered cell glioblastoma therapeutics [63–66]. Mesenchymal stem cells can be isolated from multiple tissues, including bone marrow and adipose tissue, with the latter being a very accessible and abundant source. Unmodified mesenchymal stem cells may have activity on their own against glioblastoma, although the data on this are not entirely consistent, possibly due to differences in mesenchymal stem cell sources and preparation methods (reviewed in [67]). Engineered mesenchymal stem cells have been made that express a broad range of proteins with potential anticancer activity and many of these have been evaluated in glioma animal models (reviewed in [67, 68]). No clinical trials for mesenchymal stem cells in glioblastoma have been completed to date; a trial of allogeneic bone marrow-derived human mesenchymal stem cells loaded with oncolytic adenovirus (NCT03896568 clinical trials.gov) is recruiting. Trials of engineered mesenchymal stem cells in other cancer types are at various stages, with one completed trial that evaluated mesenchymal stem cells expressing interferon-β in ovarian cancer (NCT 02530047). As with neural stem cells, mesenchymal stem cells are also being explored as therapeutics for multiple diseases other than cancer [69], and knowledge from these studies may help direct further research on their application to glioblastoma.

## Overview and future directions

Current clinical studies establish that cell-mediated therapies are feasible and safe for glioblastoma therapy, with clear evidence for complete response in one patient. A focus of this review is to compare the strengths and weaknesses of different starting cell types for engineered cell therapy of glioblastoma. Obviously, T cells have many positive attributes in this regard: cytotoxic T cells are one of the bodies main defenses against undesirable cells and can exhibit exquisite selectivity. Engineered T cells have been proven to be effective therapeutics in some leukemias and lymphomas [17] and the knowledge and infrastructure required for their clinical-grade production are established and expanding. It could be argued that as a starting cell for glioblastoma, the picture is less clear. While the case report described by Brown et al. [21] shows a remarkable response, much of this response was against leptomeningeal sites and disease eventually recurred in brain tissue, leading to the death of the patient. T cells are only present in very small numbers in normal brain [70] and it is possible that brain tissue has mechanisms to remove them as well as exclude them.

**Fig. 1:**
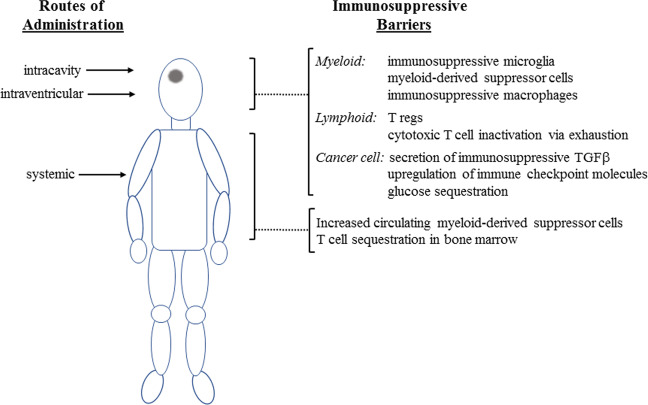
Immune barriers to engineered cell-mediated therapy of glioblastoma. Glioblastoma induces both local and systemic immunosuppression. Glioblastoma induces high levels of circulating myeloid-derived suppressor cells: these may suppress the activity of systemically administered T cells and NK cells. Glioblastoma also induces bone marrow sequestration of T cells, which could limit the engagement of endogenous T cells secondary to exogenous T-cell administration. Circulating myeloid-derived suppressor cells can be bypassed by administration into the surgical cavity or intraventricularly, but exogenously administered T cells and NK cells may still encounter local immunosuppression mediated directly by glioblastoma cells or indirectly by immunosuppressive glioblastoma-associated immune cells.

Glioblastomas have evolved elaborate multilevel defenses against T cells [71] (Fig. 1). This includes both local mechanisms and systemically active mechanisms, including T-cell sequestration in the bone marrow [72] and elevated levels of myeloid-derived suppressor cells [73]. While these systemic mechanisms can be bypassed by intracavity or intraventricular delivery, local mechanisms remain in place and may counteract the effects of T-cell therapies. Glioblastomas are infiltrated by multiple immunosuppressive cell types, including microglia, myeloid-derived suppressor cells, and macrophages, as well as regulatory T cells (Treg) [74]. Glioblastoma infiltration by macrophage-like cells was described almost a century ago [75] and can be aggressive, with these cells comprising up to 50% of cells in some glioblastoma tumors [76]. Microglia are regularly interspersed throughout normal brain parenchyma and are presumably the first immune cell type to respond. Glioblastomas induce them to adopt an activated phenotype; in cell culture, co-culture of glioblastoma cells with macrophages or microglia induces the latter to adopt an M2-like immunosuppressive phenotype, a process that is mediated by various cytokines including CSF-1, TGFβ, and GDF15 [77]. In vivo, infiltrative myeloid cells adopt a more M0-like phenotype overall [73], although the expectation is that this is still immunosuppressive. In addition to the engagement of endogenous microglial cells, glioblastomas also recruit myeloid-derived suppressor cells and macrophages from the circulation [73]. Glioblastoma tumors also have substantial Treg populations [78, 79]; these are recruited either directly via glioblastoma cell secretion of chemokines such as CCL2, or indirectly by chemokine signaling from infiltrated myeloid cells [71]. Myeloid cells and Tregs inhibit cytotoxic T cells by multiple mechanisms, including the secretion of immunosuppressive cytokines such as IL10 and TGFβ; these have multiple inhibitory effects on cytotoxic T cells including promotion of their exhaustion [80], a common state for cytotoxic T cells in the glioblastoma tumor microenvironment [81]. Beyond these indirect effects, glioblastoma cells are also able to inhibit cytotoxic T cells directly by at least three additional mechanisms: (1) T-cell inhibition by glioblastoma cell secretion of TGFβ [82]; (2) upregulation of checkpoint signals such as PD-L1 on glioblastoma cells [83, 84]; (3) aggressive glioblastoma cell competition with T cells for glucose in the tumor microenvironment [85]. Thus, while T cells have many advantages for cell-mediated therapy of glioblastoma, a complex and overlapping array of mechanisms to inhibit endogenous cytotoxic T-cell activity have evolved, many of which will be active against exogenously administered T cells.

Engineered T-cell therapy requires the use of autologous T cells, as allogeneic T-cell transplantation induces graft-versus-host disease. This is not the case with NK cells, where allogeneic transplantation is known to be safe [35]. Allogeneic transplantation may be generally preferable. This is in part because the inhibitory “self”-signal mediated by MHC can be bypassed; additionally, the use of allogeneic cells can greatly simplify manufacturing, bypassing the many issues encountered with autologous cells including patient-to-patient variability and production time. The tolerance for allogeneic transplantation also allows the use of human NK cell lines, which can further simplify production issues; however, the most widely used cell line, NK-95, was isolated from a lymphoma patient and is therefore irradiated before administration to patients. This may limit efficacy, although this could potentially be overcome with multiple dosing. Although there a several potential advantages to using NK cells over T cells, many of the mechanisms that repress T-cell activity in glioblastoma are also active in suppressing NK cell cytotoxicity; TGFβ produced by glioblastoma cells, glioblastoma-associated myeloid cells, or Tregs can repress NK cell cytotoxicity by downregulating the NKG2D activating receptor [82] and it is likely that these immunosuppressive cells also employ additional mechanisms to repress NK cell cytotoxicity. In addition, glioblastoma cells express HLA-G [86], which is known to inhibit killing by NK cells in vitro and has broad immunosuppressive activities in vivo [87, 88]. Thus, as with T cells, glioblastomas develop mechanisms to prevent their NK cell-mediated oncolysis that may also be active against exogenously administered NK cells.

Glioblastomas are very unlikely to evolve mechanisms to directly antagonize either neural stem cells or mesenchymal stem cells. However, mesenchymal stem cells do have complex and context-dependent immune functions [89] and there is evidence from other cancer types that they may be co-opted by tumors to promote immune evasion [90]. Of the four cell types discussed in this review, neural stem cells are likely to be the most “invisible” to glioblastoma, as they are normal residents of the brain and have properties in common with glioblastoma cells. However, they obviously lack the sophisticated cell-killing mechanisms present in T cells and NK cells and to date have only been engineered for relatively simple cytotoxic functions such as secretion of TRAIL or expression of pro-drug activating enzymes. Thus, the engineering problems for T and NK cells, compared to those for neural stem cells are distinct: for the former, it is overcoming evolved mechanisms that repress a sophisticated cell-killing apparatus, while for the latter it is building in an effective cell-killing function. In the long term, research in both these areas may be complementary, with contributions from both areas informing the design of effective cell-mediated therapies. While there is probably no ideal starting cell type for engineered cell therapy of glioblastoma, the good news is that there is now an abundance of tools to develop much more sophisticated biotherapeutics in this field. This includes the generation of specific human cell types through reprogramming methods, with the potential to overcome hurdles of isolating rarer cell types in amounts suitable for their use as therapeutics [91]; the use of CRISPR/Cas and related technologies to develop new cell modifications with high precision (e.g. [92]); the development of inducible systems for gene expression (e.g. [93]); ongoing improvements in protein engineering that further expand our abilities to engineer cells with properties not normally found in nature. Thus while the field of engineered cells for glioblastoma is still in its infancy, there is enormous potential for the development of new therapies that are effective against this complex and cruel disease. For engineered cell therapies that make use of antibody-targeting, the small number of quality cell surface targets is a current limitation: further detailed studies of glioblastoma expression profiles, including analyses of alternate splicing of exons [94] and microexons [95], may help overcome this. Two additional limitations to progress are the lack of high quality, clinically-relevant immunocompetent animal models of glioblastoma and the lack of good methods for in vivo imaging at single-cell resolution. Advances in these areas could facilitate more rapid preclinical and clinical evaluation of cell-mediated therapies for glioblastoma.
